# A glimpse at the aging eye

**DOI:** 10.1038/npjamd.2016.3

**Published:** 2016-03-10

**Authors:** Jonathan B Lin, Kazuo Tsubota, Rajendra S Apte

**Affiliations:** 1 Department of Ophthalmology and Visual Sciences, Washington University School of Medicine, St Louis, MO, USA; 2 Neuroscience Graduate Program, Division of Biology and Biomedical Sciences, Washington University School of Medicine, St Louis, MO, USA; 3 Department of Ophthalmology, Keio University School of Medicine, Tokyo, Japan; 4 Department of Developmental Biology, Washington University School of Medicine, St Louis, MO, USA; 5 Department of Medicine, Washington University School of Medicine, St Louis, MO, USA

## Abstract

Extensive investigations have demonstrated that organismal aging is associated with tissue dysfunction in many organs. The eye is no exception to this rule. Under healthy conditions, the eye is designed like an advanced camera with the central role of translating light from the external world into a coherent neural signal that can be transmitted to the brain for processing into a precise visual image. This complex process requires precisely maintained machinery. At the front of the eye, the transparency of both the cornea and the lens are crucial to allow passage of photons to the light-sensitive portion of the eye. Similarly, the highly organized structure of the retina located at the back of the eye is indispensable to allow for effective signal transduction and efficient signal transmission. Aging affects ocular structures in various ways, and these sequelae have been well defined as distinct clinical entities. In many instances, aging leads to ocular tissue dysfunction and disease. Nonetheless, despite clear evidence that age-associated visual impairment has significant psychosocial consequences, current treatment paradigms for many of these conditions are inadequate. In addition, strategies to decelerate or reverse age-associated deterioration in ocular function are still in their infancy. This review focuses on the cellular and molecular pathophysiology of the aging eye. Ultimately, we hope that a refined understanding of the aging eye can guide targeted therapies against cellular aging and disease.

## Introduction

**Figure 1:**
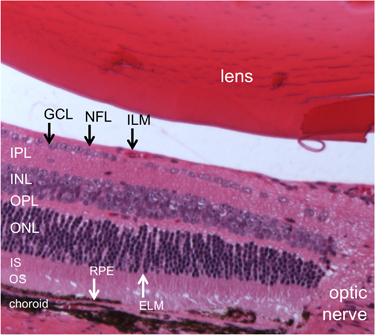
Glutaraldehyde-/formalin-fixed mouse retina stained with hematoxylin and eosin (HE) depicting the layers of the retina and other ocular structures: lens, inner limiting membrane (ILM), nerve fiber layer (NFL), ganglion cell layer (GCL), inner plexiform layer (IPL), inner nuclear layer (INL), outer plexiform layer (OPL), outer nuclear layer (ONL), external limiting membrane (ELM), inner segments (IS) & outer segments (OS) of the photoreceptors, retinal pigment epithelium (RPE), choroid and optic nerve.

Advancements in modern medicine have contributed to a marked increase in average life expectancy (lifespan) in recent decades. Nonetheless, the healthy aging process (healthspan) is often accompanied by numerous instances of age-associated dysfunction, which affect a broad range of organs, including the eye. The eye is composed of highly specialized tissues that must each maintain precise function to preserve vision. The eye is built much like a camera: light enters the front of the eye through a clear structure known as the cornea. After passing through the aqueous humor, light passes through the pupil (the dark central part encircled by the colored iris) before being focused by the crystalline lens. The light must then pass through the vitreous humor before contacting the first light-sensitive neurons of the retina, the rod and cone photoreceptors. From there, the neural signals are passed to secondary neurons (bipolar cells) and tertiary neurons (retinal ganglion cells; RGCs) with modulation from intervening neurons (horizontal and amacrine cells) before coalescing into the optic nerve (cranial nerve II) to be sent to the visual cortex of the brain. A hematoxylin and eosin (HE)-stained mouse retina, which is very similar to human retina, is presented in Figure 1 to demonstrate the highly organized structure of this organ. Even minor age-associated deviations from normal function at any step of this process can have tremendous consequences on visual function.

For example, one debilitating complication of aging is age-related macular degeneration (AMD), a leading cause of blindness in adults over 50 years of age. This disease causes deterioration of the central retina—known as the macula—and consequently, loss of the most precise central vision. Moreover, lens fibers and zonules stiffen with age, resulting in loss of accommodation and difficulty seeing nearby objects (clinically known as presbyopia). Finally, proteins in the lens called crystallins lose transparency over time, leading to cataracts, a major cause of blindness worldwide. Research suggests that oxidative stress may have an important pathogenic role in the development of senile cataracts.^1,2^

However, the detrimental effects of aging are not limited to the retina and the lens; they also affect the cornea, the ocular surface and ocular adnexa. The cells of the corneal endothelium are terminally differentiated and responsible for maintaining the cornea’s structural integrity. Although the aging process involves some loss of corneal endothelial cells over a lifetime, an accelerated loss of endothelial cells, as observed in Fuchs’ endothelial cell dystrophy, is associated with corneal edema and bullous keratopathy.^3^ Although Fuchs’ dystrophy has been associated with mutations in numerous causal genes, research suggests that they are linked by the fact that they all reduce the ability of endothelial cells to handle oxidative stress, thereby leading to accelerated cell death.^4–9^ Thus, targeted pharmacotherapy to enhance antioxidant capacity and reduced exposure to ultraviolet (UV) light to minimize UV light-induced oxidative stress are being investigated as potential therapeutic approaches. Even iatrogenic bullous keratopathy has an age association,^3^ as older patients are more likely to undergo ocular surgery, often to treat another age-associated condition.

Because it is continually exposed to UV light, the ocular surface, including the corneal and conjunctival epithelium, is also susceptible to the detrimental effects of oxidative stress.^10,11^ Aging of the ocular surface can lead to pterygium, a benign tumor on the corneal limbus, or conjunctival chalasis, loosening of the conjunctiva.^12,13^ Ocular adnexa, such as the lacrimal and meibomian glands, are also significantly affected by aging, resulting in tear deficiency and dry eye syndrome. Epidemiological studies have reported that the prevalence of dry eye syndrome increases with age.^14^ The disease affects at least 14% of individuals over the age of 50 in the United States,^3,15^ significantly reducing these patients’ quality of life and generating an enormous socioeconomic burden from the perspective of healthcare costs and lost productivity.

**Figure 2:**
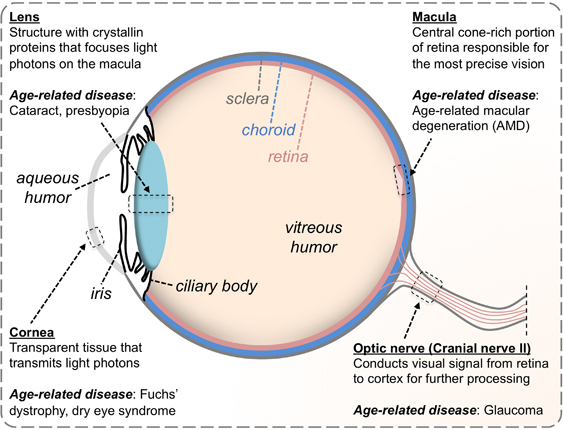
Major age-associated ocular diseases and the structures that they affect.

A summary of the major age-associated ocular diseases is presented in Figure 2. Collectively, age-associated eye disease causes visual impairment in a substantial population: estimates of the prevalence of vision impairment in adults over the age of 65 range from 4% to as high as 20%, depending on how impairment is defined.^16^ Elderly patients with low vision commonly report reduced quality of life, symptoms of depression and feelings of anxiety,^16–18^ highlighting the importance of developing more effective therapies for age-associated eye disease. This review elaborates on how the eye changes during the aging process. We hope that continued research in these areas will uncover the mechanisms that underlie age-associated ocular dysfunction and, ultimately, lead to novel targeted therapeutic approaches to delay or to prevent these sequelae and promote ‘productive aging.’

## The aging retina

The neurosensory retina is a highly organized, light-sensitive structure located at the posterior pole of the eye that is responsible for transducing visual input into neural signals to be sent to the brain. Because of their function, retinal cells are exposed to a large amount of light throughout their lifetime, making them vulnerable to light-induced damage. Psychophysical studies have reported age-associated declines in visual acuity,^19^ color perception^20^ and dark adaption thresholds.^21^ Moreover, functional testing has revealed that scotopic and photopic sensitivity (i.e., sensitivity under low light and well-lit conditions, respectively) worsens in a linear fashion during the course of adulthood.^22^

Retinal function can be tested with electrophysiological tools such as electroretinography (ERG). ERG non-invasively measures the retina’s precise electrical response to varying flashes of light with electrodes placed on the ocular surface. Different components of the characteristic ERG response correlate with the function of distinct retinal structures. Researchers have used ERG to identify age-associated declines in the amplitudes of outer retina-generated a-waves and inner retina-generated b-waves,^23,24^ along with increased b-wave implicit times.^23^ In addition, recent studies have reported reductions in the amplitudes of rod- and cone-driven oscillatory potentials by the age of 40, which may precede the gross changes in a- and b-wave amplitudes.^25^ Finally, the elderly have slower macular recovery after light stress, potentially due to reduced efficiency of photopigment restoration.^26^

These functional changes have also been correlated with various age-associated structural changes. For example, aging causes loss of retinal neurons, including rod photoreceptors,^27^ RGCs^28,29^ and rod bipolar cells.^30^ Similarly, other non-neural cells, such as retinal pigment epithelium (RPE) cells, appear to decrease in density over the course of a lifetime.^29^ Beyond loss of retinal cells, aging is also associated with accumulation of both intracellular and extracellular deposits. Intracellularly, lipofuscin, also commonly found in other organs, deposits in the RPE, where it can generate reactive oxygen species after exposure to oxygen and light.^31,32^ Recent studies have suggested that various components of these heterogeneous lipofuscin deposits may drive immune dysregulation via monocyte and microglial activation.^33^ Extracellularly, aging also leads to thickening of the acellular lamina between the RPE and the underlying choriocapillaris (also known as Bruch’s membrane; BM). In addition, there is an increase in basal laminar and basal linear deposits in BM with aging. At the transition between aging and disease, there is accumulation of esterified and unesterified cholesterol-rich material known as drusen between the RPE and BM.^34,35^ Importantly, the presence of many large drusen in the macula is the *sine qua non* of nonexudative or dry AMD.

## Age-related macular degeneration and the immune system

**Figure 3:**
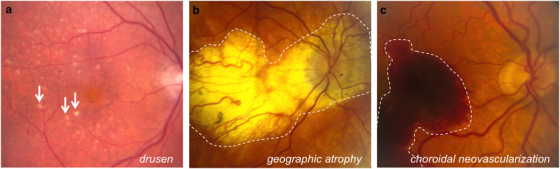
Fundoscopic images from AMD patients demonstrating hallmarks of disease, such as drusen (**a**; examples indicated by arrows), geographic atrophy (**b**; roughly outlined by dashed white line) and choroidal neovascularization (**c**; roughly outlined by dashed white line).

AMD is a complex multifactorial disorder and a leading cause of blindness in adults over the age of 50. Early AMD is characterized by the presence of lipid-rich deposits (drusen) in the subretinal space. Progression of disease can manifest in two forms: dry AMD, characterized by geographic atrophy (GA), and wet AMD, characterized by choroidal neovascularization (CNV). Both types can lead to vision loss. Fundus photographs depicting drusen, GA and CNV are presented in Figure 3. Among other contributors, the immune system has a crucial role in AMD pathogenesis. For example, a common variant in the complement factor H (*CFH*) gene (i.e., Tyr402His) confers a significantly increased risk of developing AMD, as the variant CFH protein has a reduced ability to regulate the alternate pathway of complement activation.^36–38^

Oxidative damage and inflammation also play important roles in disease progression in dry AMD. AMD donor eye tissues have been shown to contain more carboxyethylpyrrole (CEP)-adducted proteins in the outer retina compared with donor eye tissues from healthy controls,^39^ and a follow-up study in a mouse model demonstrated that these CEP-modified proteins may contribute to the development of RPE lesions mimicking GA through immune-mediated damage.^40^ Other groups using mouse models have found that subretinal infiltration of proinflammatory M1 macrophages^41^ and antigen-specific T cells activated by oxidative damage^42^ may also have a role in causing RPE death. Finally, age-associated DICER1 deficiency in the RPE of patients with GA leads to accumulation of repetitive element-derived *Alu* RNA transcripts,^43^ which leads to inflammasome activation in a mouse model and may thereby contribute to the pathogenesis of dry AMD.^44^

In contrast, many research efforts on the pathogenesis of wet AMD have focused on the role of macrophage aging. Studies involving mouse models have revealed that classically activated, M1-like macrophages tend to be anti-angiogenic.^45^ However, as macrophages age, they tend to polarize to an alternative, M2-like phenotype with an altered cytokine profile.^46^ This alteration contributes to aberrant inflammation and the inability to inhibit abnormal angiogenesis,^46^ thereby permitting CNV in advanced wet AMD and causing vision loss. Old macrophages also exhibit impaired cholesterol efflux, leading to dysregulated inflammation and pathologic vascular proliferation.^47^ The interaction between macrophages and the lipoproteinaceous drusen found in AMD patients^48^ may promote abnormal macrophage activation.^47^ Recent studies also suggest that both the rho-associated, coiled-coil-containing protein kinase (ROCK)^49^ and the IL10-driven STAT3 signaling pathways^50^ may drive aging-dependent alternate activation of macrophages.

Aberrant activation of the renin–angiotensin system (RAS) has also been shown to have a pathogenic role in the development of CNV in the setting of wet AMD. Using the laser-induced mouse model of CNV, Nagai *et al*.^51^ showed that treating mice with the angiotensin II type 1 receptor (AT1R) antagonist telmisartan reduced CNV volumes, perhaps by blocking downstream AT1R-mediated inflammation. A follow-up study from the same group demonstrated that this suppression of CNV could also be achieved upstream through (pro)renin receptor blockade.^52^ Taken together, these studies provide strong evidence for involvement of the RAS in the pathophysiology of wet AMD.

Other studies have focused on the multifactorial contributions to AMD pathogenesis. For example, one group demonstrated that a mouse model combining three known AMD risk factors—age, high-fat diet and a particular apolipoprotein E genotype—exhibits disease manifestations that resemble those found in AMD patients, including sub-RPE deposits and drusenoid deposits.^53^ This same group recently reported that a different mouse model combining advanced age, high-fat diet and decreased CFH also exhibits human AMD-like features.^54^ These findings confirm that the mechanisms underlying AMD pathogenesis are complex and involve both genetic and environmental factors.

Unfortunately, there is no current medical or surgical treatment for dry AMD, although randomized clinical trials have reported that oral supplementation of various antioxidants (i.e., vitamins C and E, beta carotene, zinc, lutein and zeaxanthin) may reduce the odds of disease progression.^55,56^ Currently, the main treatments for wet AMD are targeted therapies against vascular endothelial growth factor, e.g., aflibercept, ranibizumab and bevacizumab,^57,58^ or photodynamic therapy to reduce the development of abnormal blood vessels. However, these approaches treat the symptoms rather than the underlying causes of this complex disease.^59^ Further research to dissect the molecular mechanisms underlying AMD may lead to the development of much-needed novel therapies.

## Glaucoma and the future of neuroprotection

**Figure 4:**
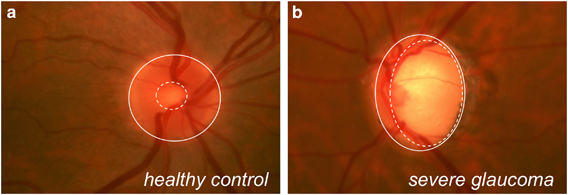
Fundoscopic image from a healthy patient with a small cup-to-disc ratio and a healthy, pink optic disc surrounding the cup (**a**) compared with a fundoscopic image from a severely glaucomatous patient with an enlarged cup and significant inferior thinning of the disc rim (**b**). Dashed white circles roughly outline the cups; solid white circles roughly outline the discs.

Age is also a significant risk factor for glaucoma, a neurodegenerative disease characterized by loss of visual fields and death of RGCs. Glaucomatous optic nerve damage can be easily visualized with biomicroscopy by examining the cup-to-disc ratio as a surrogate measure of RGC health (Figure 4). Current therapeutic strategies focus on reducing intraocular pressure (IOP), either by reducing aqueous humor production, increasing uveoscleral outflow or increasing outflow through the trabecular meshwork. Although this strategy works in some patients, other patients show disease progression despite treatment. In addition, some patients develop glaucomatous optic nerve damage in the absence of elevated IOP; this condition is clinically known as normal-tension glaucoma. Research has shown that RGC death associated with glaucoma may be mediated by numerous mechanisms, including but not limited to glutamate-mediated excitotoxicity,^60,61^ oxidative stress^62–64^ and mitochondrial dysfunction.^65^ However, most neuroprotection strategies thus far—notably including the large memantine trial—have been unsuccessful, highlighting the need for additional research to develop novel therapeutic targets.^66^

## Dry eye syndrome

**Figure 5:**
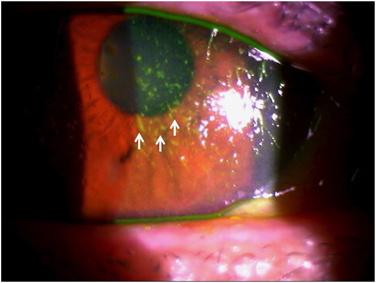
Slit-lamp photograph of a patient with severe dry eye syndrome whose ocular surface was stained with fluorescein. Note the abnormal staining on the corneal epithelium (see arrows).

Dry eye syndrome (DES) is an under-recognized health hazard, affecting millions of people in the world.^67^ Although some DES patients have a near normal-appearing ocular surface, they have an unstable tear film, which leads to symptoms such as ocular fatigue, eye irritation and blurred vision. Figure 5 shows an example of a patient with severe dry eye with abnormalities of the cornea and the conjunctival epithelium. Extensive use of portable electronic devices, such as smartphones and laptops, may contribute to the development of DES by decreasing blink rate.^68^ Tear production by the lacrimal gland also decreases with age,^69,70^ further contributing to DES, but the mechanism of this age-associated change is unknown. Similarly, the meibomian glands, which produce the lipid component of tears, are also affected by aging.^71^ One hypothesis suggests that oxidative stress may be a cause of DES. In support, several studies show that mice lacking superoxide dismutase 1 (*SOD1*) or nuclear factor erythroid 2-related factor 2 (*NFE2L2*, also known as *Nrf2*) exhibit increased oxidative stress and subsequently develop reduced tear production.^72–74^ As a result, suppression of oxidative stress with oral supplements has become an emerging strategy for treating DES.

Beyond modulating oxidative stress, altering metabolism may also be a potential strategy for DES therapy. Calorie restriction and exercise have proven to be reliable strategies for decelerating the aging process, and these approaches have also been shown to be effective in an animal model of DES.^75,76^ In support of this potential therapeutic avenue, limited human data have also shown that a sedentary lifestyle or the presence of metabolic syndrome may be related to DES.^77^ As is the case for AMD, a growing body of literature suggests that inflammation is a major contributing factor to the development of DES,^78,79^ offering another potential therapeutic target. Currently, the only approved therapy for DES is an eye-drop formulation of cyclosporine A,^80^ but alternative approaches, such as omega-3 fatty acid (EPA/DHA) supplements or agents that modulate the RAS within the lacrimal gland, are being actively investigated.^81–83^

## Circadian rhythms: the eye’s role in the body’s clock

Beyond mediating vision, the eye also has a crucial role in regulating circadian rhythms and thereby regulates broad physiological processes, such as metabolism. The recently- discovered intrinsically photosensitive RGCs (ipRGCs) transfer nonvisual information to the suprachiasmatic nucleus of the hypothalamus, the master controller of the body’s circadian rhythms.^84^ In other words, the eye both functions as a camera and sets the body’s clock.^85^

Just as aging affects the camera function of the eye, aging also affects the ability of the eye to set the body’s clock. As the lens ages, it becomes worse at transmitting short-wavelength visible (i.e., blue) light, while retaining the ability to transmit longer- wavelength visible (i.e., red) light.^86^ This effect of aging is problematic since the maximal sensitivity of ipRGCs is in the blue-light region (460–480 nm).^87^ Therefore, surgery to correct cataracts, a disease of the aging lens, is important because it restores not only visual function but also the eye’s ability to regulate circadian rhythms.^88,89^ Maintenance of proper circadian rhythms is important given its potential implications for a diverse spectrum of diseases, such as sleep disorders, obesity, metabolic syndrome, depression, breast cancer and prostate cancer.^90–93^ Of note, despite its importance for setting circadian rhythms, blue light is also hazardous to the aging retina,^94^ complicating issues with an apparent discrepancy between the demands of the eye and those of the whole body. Future research in this field is critical and will likely provide important insights into the effects of selective wavelengths of light on health and disease.

## A bright future for the eye

The aging eye and how the aging process can transition to diseases like AMD, glaucoma and DES are important issues in the field of aging research. Interventions that decelerate or reverse biological aging of the eye, such as modulation of the NAD^+^-sirtuin axis, activation of autophagy and caloric restriction, are attractive therapeutic strategies. Research focused on understanding how aging affects the eye will likely generate valuable discoveries with important clinical and day-to-day applications. Ultimately, these efforts will also lead to the discovery of common unifying pathways that drive the pathobiology of age-associated disease, both of the eye and beyond. If this goal is realized, new therapies will no longer target just one ailment but instead can target the far-reaching effects of systemic aging.
